# Recent advances in dye and metal ion removal using efficient adsorbents and novel nano-based materials: an overview

**DOI:** 10.1039/d1ra06892j

**Published:** 2021-11-11

**Authors:** Ahmad K. Badawi, M. Abd Elkodous, Gomaa A. M. Ali

**Affiliations:** Civil Engineering Department, El-Madina Higher Institute for Engineering and Technology Giza 12588 Egypt Dr.AhmedKaram91@gmail.com +20 1114743578; Department of Electrical and Electronic Information Engineering, Toyohashi University of Technology 1-1 Hibarigaoka, Tempaku-cho Toyohashi Aichi 441-8580 Japan; Chemistry Department, Faculty of Science, Al-Azhar University Assiut 71524 Egypt

## Abstract

Excessive levels of dyes and heavy metals in water sources have long been a source of concern, posing significant environmental and public health threats. However, adsorption is a feasible technique for removing dye contaminants and heavy metals from water due to its high efficiency, cost-effectiveness, and easy operation. Numerous researchers in batch studies extensively evaluated various adsorbents such as natural materials, and agriculture-derived and industrial wastes; however, large-scale application is still missing. Nanotechnology is a novel approach that has arisen as one of the most versatile and cost-effective ways for dye and heavy metal removal. Its promotion on large-scale applications to investigate technological, fiscal, and environmental aspects for wastewater decontamination is particularly important. This review critically reviews wastewater treatment techniques, emphasizing the adsorption process and highlighting the most effective parameters: solution pH, adsorbent dosage, adsorbent particle size, initial concentration, contact time, and temperature. In addition, a comprehensive, up-to-date list of potentially effective low-cost adsorbents and nano-sorbents for the removal of dyes and heavy metals has been compiled. Finally, the challenges towards the practical application of the adsorption process based on various adsorbents have been drawn from the literature reviewed, and our suggested future perspectives are proposed.

## Abstract 2

Various materials including waste precursors used as adsorbents for water treatment (dyes and metal ions removal).

## Graphical abstract

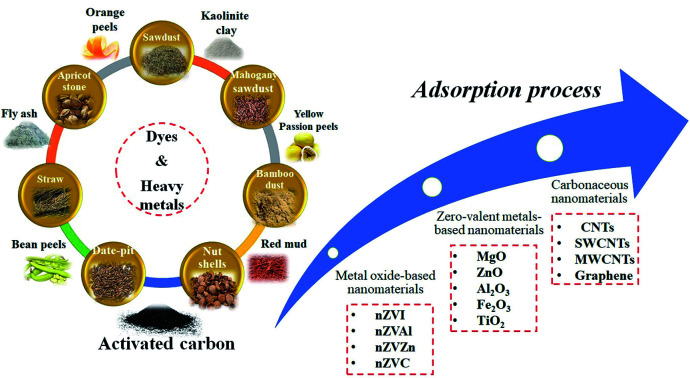

## 1. Introduction

During various human activities large quantities of fresh water are used and discarded as wastewaters containing different pollutants. A pollutant means a material/substance that alters the nature of the environment by chemical, biological or physical means, causing subsequent pollution in water, soil, and/or air. Dyes are xenobiotic and natural compounds making substances colored. Colored wastewater release comprises a public health concern as well as a serious environmental issue.^1^ More than 8000 dyes – whether insoluble or soluble – have been used and manufactured in various industries such as paper, dyeing, pulp, textile, paint, and tannery industries. Dyes are considered a pollutant because of the imparted color to water and their chemical toxicity, which is not acceptable. If these dyes are not properly handled, they can remain stable in the environment for long time, causing significant health impacts.^2^ For instance, hydrolyzed Reactive Blue 19's (RB19) half-life is about 46 years at 25 °C and pH 7.^3^ Moreover, most of them contain chromium in their molecular structure, which is carcinogenic.^4,5^ The dyes are also toxic and mutagenic in many microbiological species, as well as being teratogenic. It may adversely affect the photosynthetic activity in marine life as it reduces light penetration. Furthermore, it can cause serious side effects to human beings, such as renal failure and liver damage.^2,6^ On the other side, rapid industrialization causes excessive release of heavy metals into the environment and thus causes a global concern because of their chronic toxicity. Cadmium, manganese, arsenic, mercury, chromium, cobalt, copper, lead, iron, vanadium, molybdenum, bismuth, and nickel are often present in industrial wastewaters and originate mainly from mining activities, battery manufacture, metal plating, petroleum refining, pesticides, tanneries, smelting, pigment manufacture, paint manufacture, photographic industries, and printing.^7,8^ Heavy metals usually occur with concentrations beyond the safe permissible limits, and thus, they should be eliminated. Heavy metals can be accumulated in living organisms and are non-biodegradable, unlike organic wastes, and they could cause many disorders and diseases.^9,10^Table 1 summarizes the toxicity and the allowable limits of certain metal ions.

**Table 1:** The most common emerging heavy metals and dyes with negative effects and limits according to the World Health Organization (WHO)^11–13^

Contaminants		Health hazards	Permissible limit (mg L^−1^)
Metal ions	Cr^6+^	Headache, nausea, carcinomas, and lung tumors	0.05
	Cu^2+^	Liver damage, muscle weakness, insomnia	2.5
	Ni^2+^	Dermatitis, lung cancer, and persistent asthma	2
	Ar^3+^	Lung and kidney cancer and nausea	0.01
	Cd^2+^	Emphysema, cancer and kidney damage	0.003
Dyes	Color	—	15 Pt. Co

**Fig. 1:**
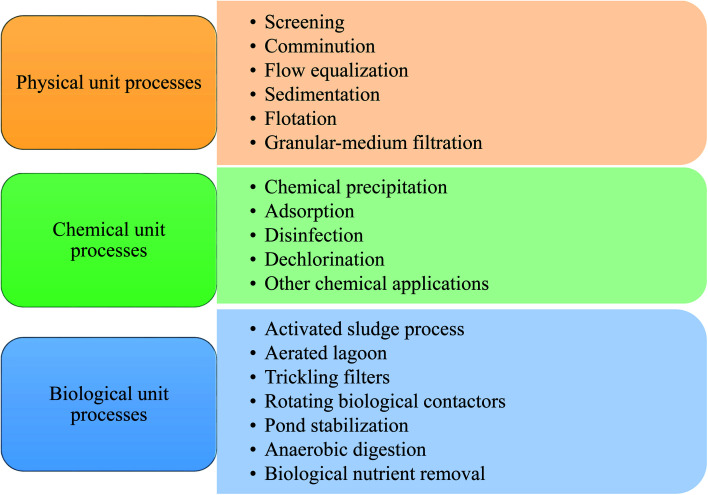
Wastewater treatment unit operations and processes.

Nthunya *et al.* evaluated the toxic metal ions levels in certain water sources in Lochiel, South Africa. It was found that some water sources contain high concentrations of toxic metal ions that exceed the WHO set limits.^14^ On the other side, significant levels of phenols and polycyclic aromatic hydrocarbons (PAHs) were also reported in the Nandoni dam, South Africa as a result of human activity in the area.^15^ Over the past decade, many operations and processes have been developed to deal with different kinds of pollution. Traditional wastewater treatment operations have been used for wastewater treatment for a long period through microbial and chemicals removal of many types of contaminants threatening the environment and public health.^16^ The various processes responsible for contaminants removal from wastewater can be classified into biological, physical, and chemical methods.^17^Fig. 1 shows the three main types: physical, chemical, and biological processes for wastewater treatment.

During physical unit operations, removing contaminants and screening large debris or solids are achieved by applying physical forces. Large floating material present in the waste flow is grinded using comminutors. Then sedimentation technique is then employed for gravitational settling of large particles suspended in the mixture.^18^

Chemical unit operations are employed to remove contaminants for wastewater treatment using chemical reaction means. Chemical coagulation of initial wastewater is involved in this kind of operation to promote sedimentation of divided solids and turn them into more settleable flocs.^19^ Thus, improving the removal efficiency of suspended solids. Soluble substance collection is then maintained within a soluble solution with a proper interface during the adsorption process. Activated carbon (AC) is one of the well-known adsorbents employed in such a process. Chemical agents like chlorine and its alternatives are then used for micro-organisms disinfection. Dichlorination occurs for chlorine residue removal from wastewater; AC may also be employed in this process. Finally, conversion of the dissolved organic and finely divided matter into flocculent inorganic or organic solids is achieved during the biological unit processes. In these processes, bacteria and other micro-organisms convert the colloidal dissolved carbonaceous and organic matter into various gases, which are then removed in sedimentation tanks.

## 2. Adsorption process and the affecting factor

The adsorption process is a chemical technique and is considered among the most promising routes for industrial wastewater treatment. The ease of operation, along with the superior ability to remove non-biodegradable contaminants, classified the adsorption process as one of the most popular wastewater treatment techniques, especially for the treatment of industrial effluents.^20–23^ Further merits of the adsorption process, such as possible economic regeneration of the adsorbent and little sludge generation, make it among the essential treatment processes for water-consuming industries, such as textile and paints industries. Extensive research has shown the efficacy of the adsorption process for textile and paints wastewater treatment.^7,21,24^ Several batch and continuous studies were performed for the adsorption of dyes and metals ions and achieved respectable contaminants deduction in a short contact time.^24,25^ Generally, the adsorption process is mainly affected by several environmental factors such as; pH, adsorbent dose, agitation rate, contact time, temperature, and the physical characteristics of the used sorbent material, including sorbent surface area and particle size.^21,26^ Edison GilPavas *et al.* reported a unique method that incorporated sequential electrocoagulation (EC) and electro-oxidation (EO) with AC adsorption to treat industrial textile wastewater.^27^ Adsorption process on AC with 1200 m^2^ g^−1^ BET surface area and 30 mesh size was employed to minimize active chlorine and other materials that lead to remained toxicity after the sequential (EC + EO) processes. Their results revealed that EC and EO achieved 88% reduction of COD, total discoloration, and 79% mineralization of TOC. In addition, more biocompatible effluent (BOD_5_/COD = 0.58) was obtained. However, the toxicity was high.

By incorporating AC adsorption, effluent toxicity was significantly reduced (*Artemia salina* mortality = 0%). While, Ayub *et al.* used EC with adsorption process to remove heavy metals such as Cr, Cu, and Zn.^8^ Their results showed that Cr, Cu, and Zn removal increased by increasing the applied electric current, the concentration of sodium chloride, and electrocoagulation time. In addition, they found that, under the optimum conditions (applied 2 A electric current, pH = 4, and 60 min of electrolysis time), 100% Cu, 99.2% Zn, and 87.6% Cr, were removed. In addition, Razali *et al.* prepared WO_3_ photocatalyst and studied its adsorption performance against palm oil mill effluent (POME) over 300 min.^28^ Adsorption results showed that the WO_3_-400 °C prepared sample possessed a relatively higher efficiency against pond aeration pollution than anaerobic and cooling ponds. Moreover, WO_3_ was able to degrade POME in darkness continuously, and color removal of about 48% was achieved.

### 2.1 Effect of pH

**Fig. 2:**
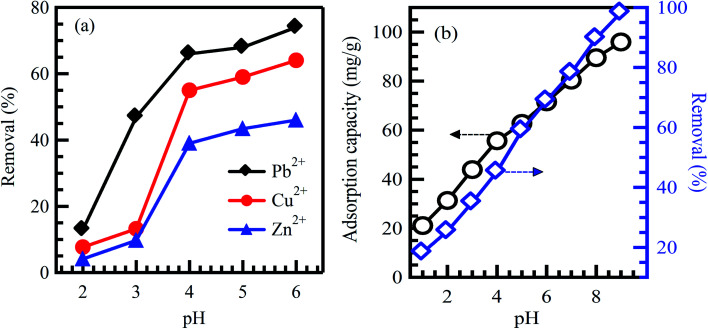
Effect of initial solution pH on the adsorption of (a) Pb^2+^, Cu^2+^ and Zn^2+^ on modified crosslinked cellulose/sodium alginate adsorbent, reproduced with permission from ref. 40, copyright, Royal Society of Chemistry, 2018; and (b) MO dye on functionalized MWCNTs, reproduced with permission from ref. 41, copyright, Springer, 2019.

pH is the measure of acidity or alkalinity of an aqueous solution. Studying pH influence is fundamental for the adsorption of dyes and heavy metals removal. The surface charges of sorbent materials and the degree of ionization of acidic and basic compounds are sensitively affected by solution pH.^29^ Therefore, the contaminants uptake rates might be enhanced or depressed based on the initial value of solution pH. As a result, the hydrogen and hydroxyl ions are adsorbed with great efficiency, which enhances the ability of the adsorption process as the functional groups of the adsorption sites disintegrate, leading to a change in reaction kinetics and equilibrium characteristics.^30^ Generally, at lower pH value, the uptake rate of dyes will be affected and decreased for cationic dyes adsorption. However, the percentage of dye uptake will be increased for anionic dyes.^31^ The crucial factor affecting surface adsorption ability and the type of surface-active centers is the point of zero charges (pH_pzc_).^7,31^ pH_pzc_ is a common way to gauge or define the electrokinetic properties by determining the pH at which the surface charge is zero. The pH value is exclusively used to represent PZC in systems where H^+^/OH^−^ are the primary determining ions. Many studies analyzed pH_pzc_ of various adsorbents made from agricultural wastes. It was reported that cationic dye adsorption is favored at pH greater than pH_pzc_ because of the presence of functional groups such as the OH^−^ group, whereas anionic dye adsorption is favored at pH less than pH_pzc_, where the surface becomes positively charged.^32,33^ Investigating the optimum pH of the solution is a continuing concern by researchers within the adsorption process. Hameed *et al.*^34^ studied methylene blue (MB) eradication from an aqueous solution based on papaya seeds adsorbent. The effect of initial pH has been conducted at pH from 3 to 10 with 50 mg L^−1^ initial dye concentration. The uptake rate of MB is observed low at pH 3 and significantly enhanced up to pH 4. It may be attributed to the positive surface charge; as a result, making (H^+^) ions in contrast with dye cations causes a decrease in the uptake rate of the dye. Similar results were stated for the adsorption of MB on grass waste,^35^ yellow passion fruit peel,^36^ and *Citrus limetta* peel.^37^ In contrast, at a high pH range, the uptake rate of dyes will be affected and increased for cationic dyes adsorption. This demonstrates that alkalinity improves the electropositive adsorption of substances where acidity decreases the adsorption of positively charged dye due to electrostatic repulsion.^31^ However, the percentage of dye uptake will be decreased for the adsorption of anionic dyes. It illustrates that the lower uptake rate of cationic dyes at acidic pH may be attributed to the excess presence of (H^+^) ions competing with the dye cation groups for adsorption sites. It was reported in ref. 38 that the maximum dye uptake for cationic dyes, C.I. basic blue 9 (BB9) and C.I. basic green 4 (BG4) were 352.76 and 293.32 mg g^−1^, respectively at pH > 5 on to anionic poly-γ-glutamic acid-based adsorbent. Similar results were reported in ref. 39 for the adsorption of basic red 2 (BR12) dye. The maximum of BR12 adsorption onto animal bone meal adsorbent was achieved at pH > 9.1. Zhan *et al.*^40^ reported that adsorption of Pb, Cu, and Zn metal ions onto modified crosslinked cellulose/sodium alginate adsorbent was increased with an increase in solution pH (Fig. 2a). Similarly, Sadegh *et al.*^41^ reported the increased adsorption rate of methyl orange (MO) onto functionalized MWCNTs with an increase in solution pH (Fig. 2b).

### 2.2 Effect of adsorbent dosage

**Fig. 3:**
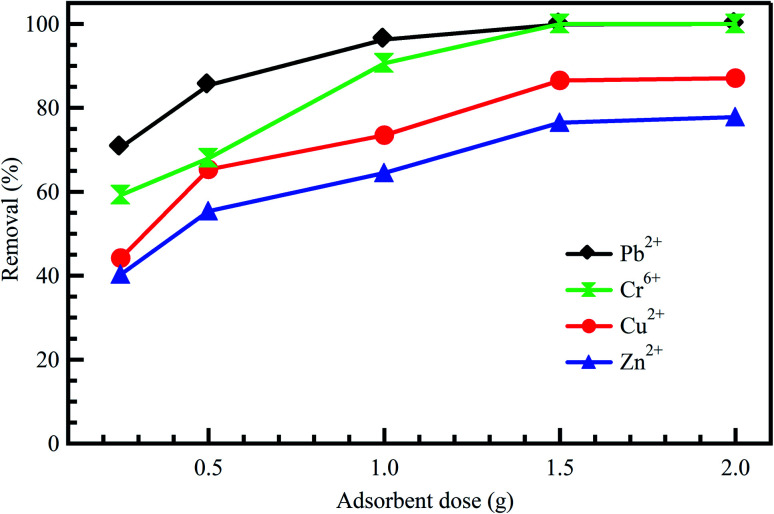
Effect of adsorbent dose on the adsorption of diverse metal ions onto palm kernel shell, reproduced with permission from ref. 43, copyright, Nature, 2019.

The effect of adsorbent dosage is an important factor to be considered as it plays a key role in improving the efficiency of the treatment process.^21^ At constant dye concentrations, the removal efficiency of the dye increases with adsorbent dose due to the availability of adsorption sites accompanying the great surface area. Accordingly, this could increase the efficiency of removing dyes and reduce the concentrations of other undesirable contaminants like heavy metals.^42^ Baby *et al.*^43^ reported that adsorption of Cr^6+^, Pb^2+^, Cd^2+^, and Zn^2+^ onto palm kernel shell adsorbent was increased with an increase in adsorbent dosage (Fig. 3). The same conclusion was reported by Li *et al.*^42^ for the adsorption of Zn^2+^, Cu^2+^, Cd^2+^, and Pb^2+^ onto Fe_3_O_4_/MnO_2_ nano-composites. On the other hand, the adsorbent dosage is particularly at the heart of applying any economic study of adsorbent per unit of wastewater to be treated. After studying the effect of adsorbents doses on the treatment process, it becomes clear that the treatment as a whole is economical, as it did not need large doses of the adsorbent material, which was expensive regarding preparation or regeneration. Ahmad *et al.*^44^ indicated a significant positive impact of AC dosage on the removal of COD and color from real textile effluent. However, by utilizing dose >0.3 gm, a slight low effect is detected on the removal of COD and color thanks to the unsaturation of adsorption sites throughout the adsorption process. Similar behavior was observed for AC resulting from coconut waste of metal ions; Pb^2+^, Hg^2+^, and Cu^2+^ removal from industrial effluents.^45^

### 2.3 Effect of contact time

**Fig. 4:**
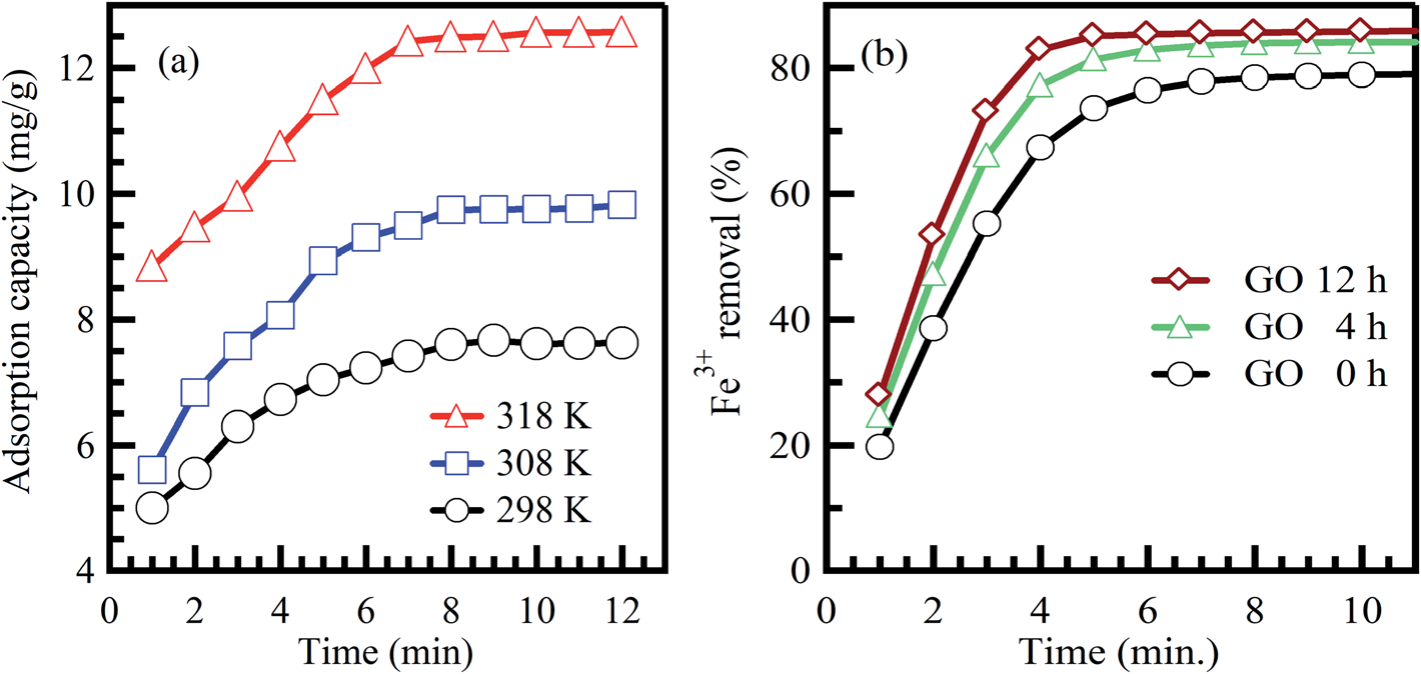
Effect of contact time on the adsorption of (a) bromothymol blue dye on polyvinyl alcohol adsorbent, adapted with permission from ref. 49, copyright, Elsevier, 2016, and (b) Fe^3+^ on graphene oxide aerogel adsorbent, adapted with permission from ref. 50, copyright, Elsevier, 2019.

Commonly, long contact time increases the adsorption capacity and contaminant removal efficiency.^46^ In the beginning, the amount of adsorbed contaminant onto the adsorbent surface rises fast, and after some time, the adsorption process decelerates down and reaches a constant value.^47^ This could be credited to the amount of the adsorbed contaminant at the state of dynamic equilibrium. The essential contact time to achieve this equilibrium state is called equilibrium time.^48^ The amount of the adsorbed contaminant at the equilibrium time imitates the extreme adsorption rate of the sorbent material under operating conditions.^47^ Agarwal *et al.*^49^ reported that the adsorption rate of bromothymol blue dye on polyvinyl alcohol adsorbent is increased effectively when the interaction time increases at different temperatures (Fig. 4a).^49^ Furthermore, Lee *et al.*^50^ reported the same conclusion for the adsorption of Fe^3+^ on graphene oxide aerogel adsorbent (Fig. 4b).^50^ In conclusion, the equilibrium state is credited to reducing adsorption rates due to the absence of available sites for contaminant adsorption (saturation state).^51^

### 2.4 Effect of initial concentration

**Fig. 5:**
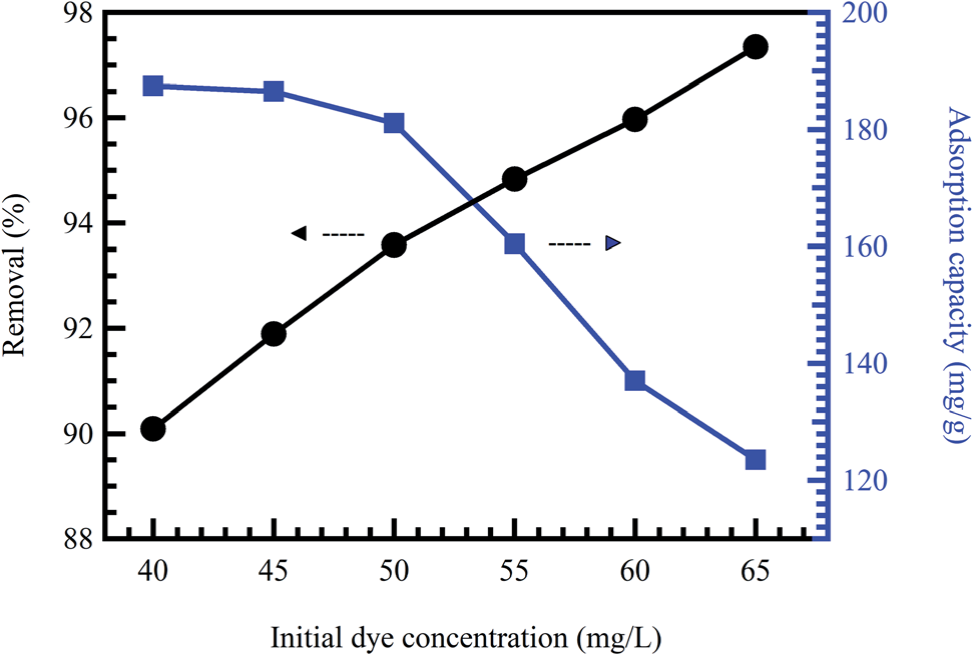
Effect of initial MB concentration onto ZnCl_2_ corn husk AC adsorbent at pH = 4, and adsorbent dose = 0.3 g L^−1^, reproduced with permission from ref. 55, copyright, Hindawi, 2013.

Initial concentration delivers a vital energetic force for improving mass transfer resistances of contaminant molecules between the solid phases and aqueous solution. Therefore, a greater initial contaminant concentration improves the adsorption rate and demands a longer equilibrium time.^31^ Commonly, at low concentrations, the ratio between the initial numbers of contaminant molecules to the accessible surface area is low. Consequently, the adsorption rate is not influenced by the initial contaminant concentration.^52^ Nevertheless, at high concentrations, the vacant adsorption sites become fewer, and therefore, the contaminant removal rates rely on the initial concentration.^53^ At constant adsorbent doses, adsorption capacity rises compared to the contaminant concentration. However, the removal percentage declines, demonstrating that residual contaminant concentration is greater than the initial concentration.^21^ Zhang *et al.*^54^ reported a reduction in the adsorption rate of MO dye, accompanied by an increment in the MO concentration from 20 to 400 mg L^−1^. Khodaie *et al.*^55^ also reported the same behavior for the adsorption of MB dye onto ZnCl_2_ corn husk AC (Fig. 5).

### 2.5 Effect of temperature

**Fig. 6:**
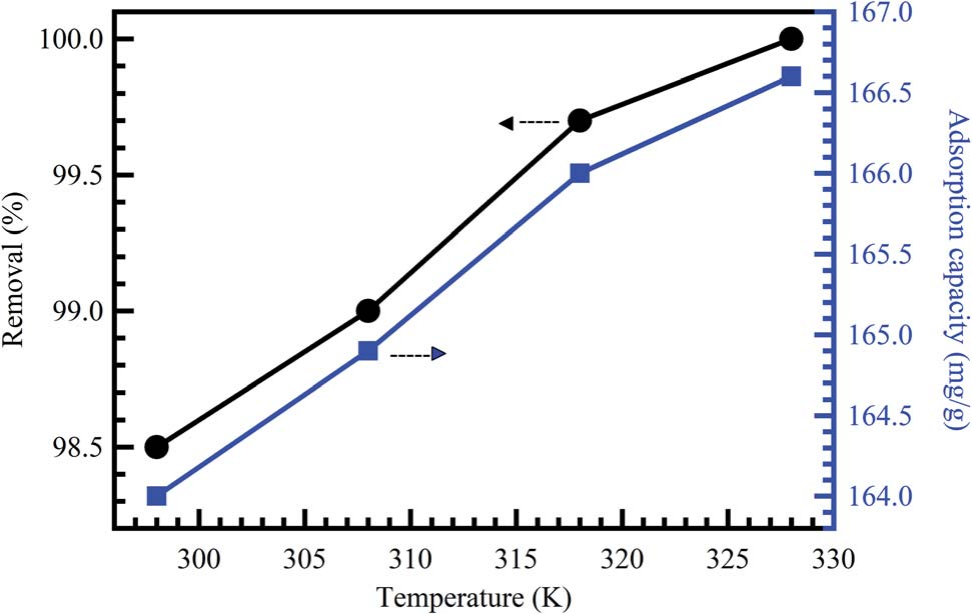
Effect of temperature on the adsorption of MB dye onto activated corn husk carbon, reproduced with permission from ref. 55, copyright, Hindawi, 2013.

Temperature change has a chief impact on the adsorption rate as it can progress the diffusion rate of the adsorbent molecules through the external layer and the inner pores of the sorbent material particles, thanks to the lessening in the solution viscosity.^56^ Furthermore, temperature change could also raise the tendency of deaggregation of the adsorbent for a particular adsorbate. Generally, at elevated temperatures, the equilibrium of the adsorption process could be reduced, demonstrating the adsorption reaction's exothermic nature. The high rise in temperature could moreover cause an upsurge in the kinetic energy among contaminant molecules^57^ and adsorbent particles, the result of the advanced collision rate between sorbent material and contaminant molecules.^58^Fig. 6 shows the effect of temperature on the adsorption of MB dye onto activated corn husk carbon.^55^ The results demonstrated that dye adsorption is temperature dependent. The mobility of the dye molecule increased as temperature increased. This means that the dye molecule efficiently interacts with the absorbent material when the surface temperature is raised. In conclusion, temperature could mark the adsorbent's chemical potential and could affect the desorption step and, as a result, affect the adsorption equilibrium reversibility. Many works have been conducted to determine the influence of solution pH, contact time, adsorbent dose, initial concentration, and temperature on dyes and metal ions adsorption, as summarized in Table 2.

**Table 2:** The effect of diverse operating parameters on dye and metal ion removal by various adsorbents

Contaminant		Adsorbent	pH	Adsorbent dosage	Contact time (min)	Initial concentration (mg L^−1^)	Temp. (°C)	Adsorption capacity (mg g^−1^)	Ref.
Dyes	MG	Peroxide treated rice husk	11	0.2 g/100 mL	45	30	30	26.6	59
	MB	Pine leaves	9.2	15 mg/50 mL	100	10	30	126.58	60
	Brilliant green	NaOH treated sawdust	2.9	4 g L^−1^	180	200	30	46.51	61
	Crystal violet	Kaolin	7	1 g L^−1^	5	20	22	45	62
	Lavfix fast rad	MgO	6	25 mg/100 mL	45	25	25	92.16	63
Heavy metal ions	Cr^6+^	CuO	3	1 g L^−1^	10	20	25	13.1	64
	Pb^2+^	MNCPs	5	7.5 mg L^−1^	15	10	25	53.33	65
	Ag^2+^	MnO_2_ nanotubes@rGO hydrogel	5	0.2 g L^−1^	120	200	25	138.2	66
	Zn^2+^	MnO_2_ nanotubes@rGO hydrogel	5	0.2 g L^−1^	120	200	25	83.9	66
	Cr^3+^	Treated orange peel	3	0.1 g/100 mL	120	10	25	9.43	57
	Fe^3+^	Pretreated orange peel	3	0.1 g/100 mL	120	30	25	18.19	57
	Cu^2+^	Fe_3_O_4_	7	10 mg/100 mL	30	2	25	14.18	67

## 3. Synthesis and properties of adsorbent materials

### 3.1 Carbon nanomaterials

#### 3.1.1 Activated carbon

**Fig. 7:**
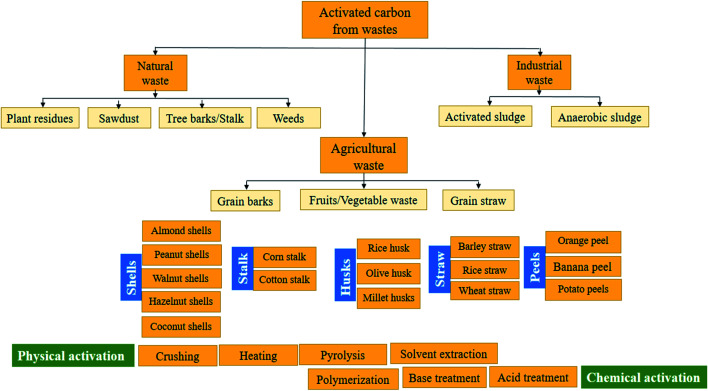
Different sources and methods used for the extraction of AC.

Activated carbon is a common carbon material used for wastewater treatment due to its large surface area.^21^ Recently, a lot of efforts were directed to investigate more economical ways of getting AC, to replace the commercial one. Several researchers investigated the properties and applications of AC extracted from various wastes such as; straw,^68^ oil palm fiber,^69^ sunflower seed hull,^70^ brasiliensis seed coat,^52^ coir pith,^71^ palm kernel shell,^72–74^ and date-pit.^75^Fig. 7 summarizes different methods and sources used for the extraction of AC.

Ahmad *et al.* used the phosphoric acid-assisted chemical activation method to prepare AC from bamboo waste for color reduction and COD of a real textile mill effluent.^44^ In a typical procedure, the raw material (40 g) was mixed with a certain volume of concentrated phosphoric acid (40 wt%) under occasional stirring. Then, impregnated samples were dried for 3 days under sunlight. After that, precursor activation was performed for 2 h at 500 °C under pure N_2_ gas flow (150 cm^3^ g^−1^) with 10 °C min^−1^ heating rate in a tubular furnace. After activation, samples were left to cool down to room temperature, followed by washing many times with hot distilled water (D.W.) (70 °C) until pH reached 6–7. Finally, at 110 °C for 24 h, samples were dried and then stored in plastic containers. They got AC maximum yield value of nearly 30.213%. In addition, the extracted mesoporous AC material possessed a BET surface area of about 988.23 m^2^ g^−1^, 0.69 cm^3^ g^−1^ total pore volume, and an average pore diameter of 2.82 nm.

Activated carbon possesses unique properties such as large surface area and great ability to remove both organic and inorganic contaminants. The efficiency of the adsorption process based on commercial AC (CAC) has been extensively evaluated to remove dyes and metals ions from industrial effluents.^7,21^ Gomez *et al.*^56^ assessed the removal of various toxic acid dyes (acid orange 61, acid red 97, and acid brown 425) onto CAC from individual solutions and in mixtures. It was revealed that CAC is efficient for acid dyes removal at different contact times from 0 to 250 min. Activated charcoal has been proficiently evaluated as an adsorbent to eradicate several toxic dyes (bromophenol blue, alizarine red-S, methyl violet, phenol red, malachite green (MG), erichrome black-T, and MB) from aqueous solutions.^76^ The impact of pH, contact time, and temperature on the adsorption performance has also been considered. It was concluded that the adsorption of all the dyes on activated charcoal declines at high pH and elevated temperatures. The great surface area is a fundamental property of CAC and its porous structure, enhancing the adsorption capacity of extensive types of pollutants from effluents.^77^ Nevertheless, the uneconomical characteristic is one of the most frequently stated problems by using CAC in industrial wastewater treatment along with the regeneration of saturated carbon is also costly and not straightforward.^21^Table 3 summarizes a non-exhaustive list of CAC and its efficiency in removing various dyes and metal ions.

**Table 3:** Adsorption capacities of CAC for various dyes and heavy metal ions removal

Contaminant		Adsorption capacity (mg g^−1^)	Ref.
Dyes	MO	35.43	53
	MB	100.00	68
	Remazol red B	144.92	78
	MB	199.60	53
	Reactive red 120	267.20	79
	CR	300.00	80
	Reactive violet 5	517.10	81
Heavy metal ions	Cr^6+^	4.72	77
	Pb^2+^	5.95	82
	Cd^2+^	10.30	83
	Cr^6+^	15.47	84
	Cd^2+^	90.09	85

#### 3.1.2 Carbon nanotubes

**Fig. 8:**
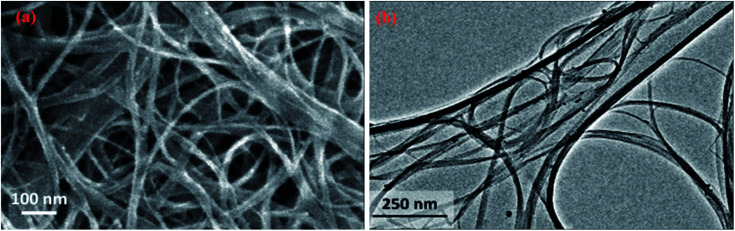
SEM (a) and TEM (b) images showing the morphology of SWCNTs, adapted with permission from ref. 91, copyright, Elsevier, 2016.

**Fig. 9:**
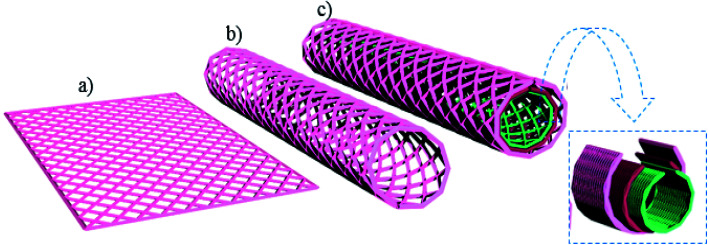
Structure representations of (a) ideal graphene sheet, (b) SWCNT, and (c) MWCNTs.

Carbon nanotubes (CNTs) have received considerable attention not only due to their large surface area but also for their outstanding mechanical, thermal, and electrical properties.^25,86^ Carbon nanotubes can be mainly divided into single-walled CNTs (SWCNTs) multi-walled CNTs (MWCNTs), as shown in Fig. 8. There are many methods used to prepare CNTs, including green methods.^87^Table 4 summarizes different routes of preparation of CNTs-based materials, along with their properties. The last decades have seen a rising development concerning CNTs thanks to their superior electrical, mechanical, thermal, and optical properties, along with great surface area and fast kinetics.^25,86,88,89^ Mainly, CNTs are graphene sheets rolled up in tubes and have two types, either single-walled CNTs (SWCNTs) as shown in Fig. 9 or multi-walled CNTs (MWCNTs) as shown in Fig. 8 and 9.^86,90^Table 4 summarizes the properties of CNTs with both types.

**Table 4:** Recent reports on the various preparation routes of CNTs-based and their properties

CNTs-based materials	Type of CNTs	Preparation method	Diameter (nm)	BET surface area (m^2^ g^−1^)	Length (nm)	Ref.
Bare CNTs	MWCNTs	Ultrasonic atomization and heat treatment	5–10	113.14	1000	92
CNTs/poly(etheretherketone)	MWCNTs	Coprecipitation injection molding	200	—	10 000–30 000	93
CNTs/Cu	MWCNTs	Alloying method	15–20	—	150–300	94
CNTs	—	Methane chemical vapor depositions	40–60	49.9–128.5	—	95
CNTs	SWCNTs	Electric arc method	20	—	700	96
Polymer/CNTs	MWCNTs	Mechanical mixing process	10–15	—	100–1000	97

**Fig. 10:**
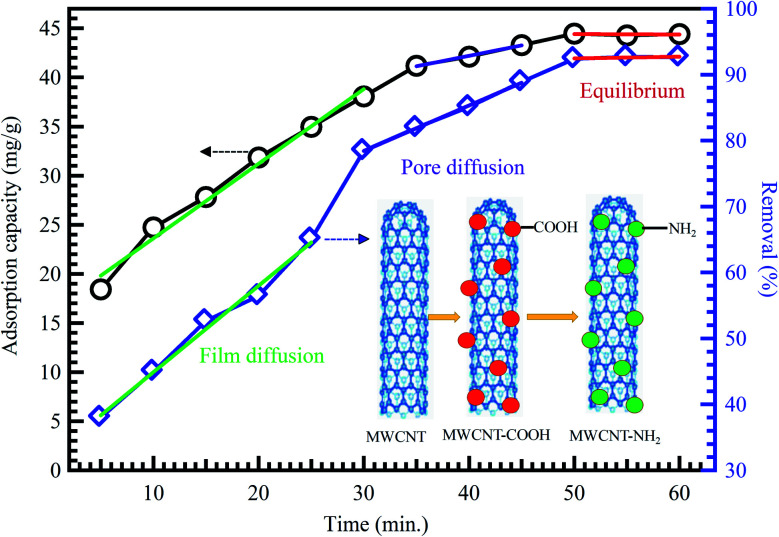
Adsorption of methyl orange on MWCNTs, adapted with permission from ref. 41, copyright, Springer, 2019.

Extensive research has shown that CNTs have a massive absorptive capacity to adsorb extensive ranges of pollutants such as heavy metals and dyes.^98,99^ Zeng *et al.*^100^ investigated the removal of organic dyes (MG) onto entangled CNTs as porous material to improve the adsorption process. The composites acquired over the polymerization with polyaniline (PANI) influenced bulky surface areas. The CNT/PANI composites revealed a 15% advanced adsorption rate of 13.95 mg g^−1^ than PANI at an initial dye concentration of 16 mg L^−1^. In another study, MWCNTs were also reported as an effective nanocomposite beads supported by chitosan for the removal of nitrates (NO_3_^−^) from water reached 96.8% for a 50 mg L^−1^ NO_3_^−^ water solution.^101^ The adsorption capacity of CNTs is highly dependent on the porosity and surface functionalization, as shown in Fig. 10.^41^

**Fig. 11:**
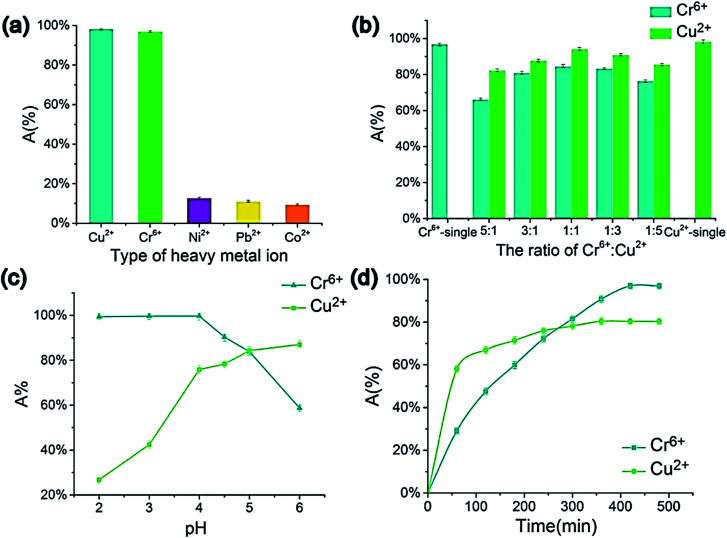
Impacts of metal ions' type (a) Cr^6+^ to Cu^2+^ ratio, (b) pH, (c) adsorption time, and (d) on the metal ions' adsorption efficiency (composite tablet adsorbed with 10 mg L^−1^ concentration under 40 °C temperature for 600 min in 4.5 pH, 5 mL of metal ion aqueous solution (1 mg L^−1^), 130 rpm), adapted with permission from ref. 104, copyright, Elsevier, 2021.

Atieh *et al.*^102^ suggested eliminating Cr^6+^ ions onto CNTs supported by AC from contaminated water. The achieved maximum adsorption capacity on AC-CNT coated adsorbent was recorded as 9.0 mg g^−1^. As a result, it seems that AC-CNT coated adsorbent is of utmost effectiveness for chromium ions removal. CNTs coated by manganese oxide (MnO_2_/CNTs) were utilized to eradicate Pb^2+^ ions from an aqueous solution.^103^ It was stated that the Pb^2+^ removal rate reduced with the decline of pH. The maximum adsorption capacity was 78.74 mg g^−1^, compared with not coated CNTs throughout the initial 15 min. For the adsorption of heavy metals and tetracyclines by MWCNTs, Chen *et al.* prepared a composite tablet based on ionic liquid-MWCNTs (IL-MWCNTs).^104^ It was found that benzothiazole ionic liquid (*N*-butyl benzothiazole hexafluoroborate, [C_4_Bth][PF_6_]) is selective for these pollutants, as a result, it was loaded into MWCNTs before tableting. Their results showed that the adsorption efficiency of TCs, Cr^6+^ and Cu^2+^ could reach 99.76, 94.10, and 84.60%, respectively, by one tablet, as shown in Fig. 11. In addition, the prepared tablet could be reused, with adsorption efficiency exceeding 90%. The adsorption capacity of CNTs on the removal of dyes and heavy metals is listed in Table 5.

**Table 5:** Adsorption capacity of different CNTs adsorbents for dyes and heavy metal ions

Adsorbent	Contaminant	Adsorption capacity (mg g^−1^)	Ref.
MWCNTs	MO	25.73	41
MWCNTs	Sufranine O	43.48	105
MWCNTs	Procion red MX-5B	44.68	106
MWCNTs	Reactive blue 4	502.5	107
SWCNTs	Reactive blue 4	567.7	107
MWCNTs	Pb^2+^	4.00	108
MWCNTs	Ni^2+^	7.53	109
SWCNTs	Ni^2+^	9.22	109
SWCNTs	Zn^2+^	43.66	110
CNTs	Pb^2+^	49.95	111

#### 3.1.3 Graphene and its derivatives

Due to graphene's outstanding physical, mechanical, and thermal properties and its derivative materials such as graphene oxide (GO) and reduced graphene oxide (rGO), they are extensively employed in many environmental applications.^112^ Several methods can prepare 3D graphene, including colloidal-sphere based, ice-templating, hydrothermal template, carbonization of polymer frameworks, lithographical template synthesis, hydrothermal reduction induced assembly, chemical reduction induced assembly, cross-linking induced assembly, filtration assisted assembly, centrifugal vacuum evaporation, sugar blowing, chemical vapor deposition (CVD).^113^

Jameel *et al.* reported the detailed methods for the preparation of rGO from graphite. Firstly, to isolate graphite, graphite from several pencils was extracted after removing the wooden part. Then, it was refined using a pestle (mortar) after polishing it from any remaining wood residues. Secondly, a modified Hummers' method was used to prepare GO.^114^ Finally, rGO was prepared from graphene oxide as the following, prepared graphene oxide (0.3 g) was added to (50 mL) freshly prepared lemon peels solution (5%) (reducing agent), then the mixture was heated at 55 °C for (2 h), graphene oxide color changed from greenish-yellow to greenish indicating the formation of rGO.^115^

**Fig. 12:**
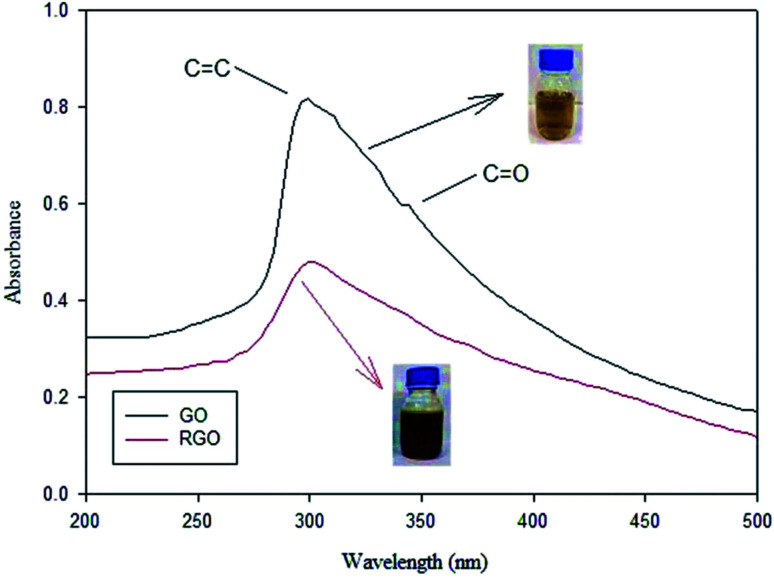
UV-Vis spectra of GO and rGO, adapted from ref. 116, copyright, Nature, 2020.

In addition, green reduction of GO into rGO was presented by Wijaya *et al.*, using the peel extract of kaffir lime (*Citrus hystrix*).^116^ Both GO, and kaffir lime peel extract with different (v/v) ratios were mixed and stirred at (600 rpm) for 8 h. Then, mixtures were washed well and sonicated for 30 min, until a clear solution was obtained. Finally, the resultant materials were dried using a vacuum oven. UV-Vis analysis was used to confirm the reduction of GO into rGO, as shown in Fig. 12. The GO transformation into rGO was revealed by the loss of the shoulder peak at ∼343 nm representing n → π* for the C=O group. In addition, GO color changes from brown to blackish after reduction *via* the peels extract of kaffir lime.

**Fig. 13:**
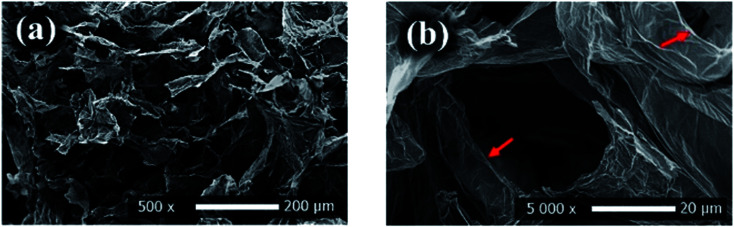
SEM images (a and b) of rGO showing its unique layered structure, adapted with permission from ref. ^117^, copyright, American Chemical Society, 2021.

Graphene and its derivatives are alternative carbonaceous sorbent nanomaterials, which are a type of one or numerous atomic layered graphite, offers exceptional two-dimensional structure as shown in Fig. 13a and b.^117^ Qian *et al.* reported the preparation of lignin–poly(*N*-methylaniline)–rGO hydrogel for both lead ions and MB dye removal.^118^ Obtained adsorption capacity of about 753.5 and 201.7 mg g^−1^ were obtained for Pb^2+^ ions and MB, respectively.

**Fig. 14:**
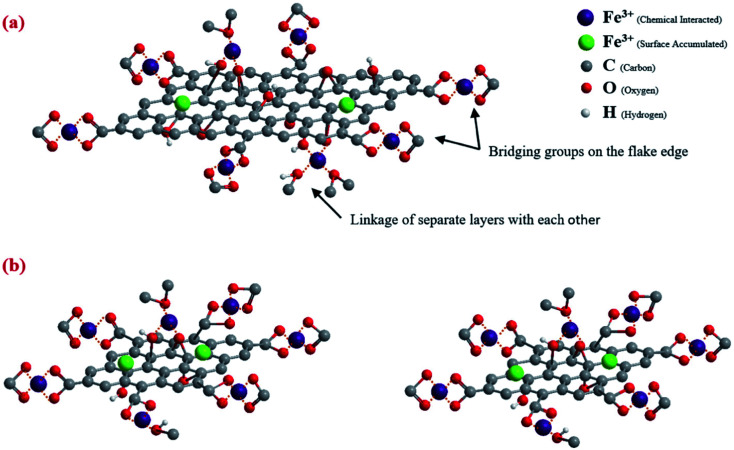
Fe^3+^ ions adsorption at GO aerogel with (a) larger flakes size (b) smaller flakes size, adapted with permission from ref. 50, copyright, Elsevier, 2019.

Zhao *et al.*^119^ synthesized graphene oxide nanosheets as sorbent materials for Cd^2+^ and Co^2+^ ions removal from aqueous solution. The reported results revealed the reliance of heavy metal ions sorption on solution pH, ionic strength, and the abundant surface functional groups of the graphene oxide nanosheets. Also, Chandra *et al.*^120^ prepared magnetite graphene sorbent material possessing about 10 nm particle size. Great adsorption capacities were reported for As^3+^ and As^5+^, indicating the great adsorption capacity attributable to the improved adsorption sites in the graphene composite. In addition, Lee *et al.*^50^ studied the effect of flake size graphene oxide aerogel on Fe^3+^ adsorption and they found that capacity of 133.3 mg g^−1^ was obtained.^50^Fig. 14 shows how Fe^3+^ ions binded to graphene oxide aerogel with different flake sizes.^50^

**Fig. 15:**
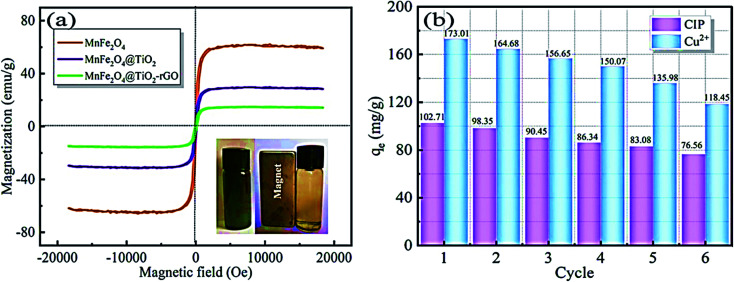
(a) Magnetic properties of the prepared composite, (b) reusability of the prepared composite for the adsorption of CIP and Cu^2+^, adapted with permission from ref. 122, copyright, Elsevier, 2021.

Reduced graphene oxide/NiO nanocomposites showed a good adsorption behaviour of Cr^6+^ of 198 mg g^−1^.^121^ While Chang *et al.* reported preparing a recyclable MnFe_2_O_4_@TiO_2_ core–shell magnetic composite loaded onto rGO as a unique adsorbent composite for ciprofloxacin Cu^2+^ removal from water.^122^ Under acidic conditions, the prepared composite showed good insolubility. Their results recorded a maximum adsorption capacity of 122.87 and 225.99 mg g^−1^ for CIP and Cu^2+^, respectively. In addition, adsorption profiles followed the Langmuir isotherm and pseudo-second-order kinetics. After 6 times of recycling, 76.56 and 118.45 mg g^−1^ adsorption capacities for CIP and Cu^2+^, respectively, were obtainable, as shown in Fig. 15.

### 3.2 Low-cost adsorbents

Recent developments in the adsorption process have heightened the need for studying the feasibility of utilizing effective economic sorbent materials. As a result, a greater focus was given to investigate economical ways of getting AC to replace the commercial one.^72,74,123^ Several researchers examine AC extracted from various wastes such as; straw,^68^ oil palm fiber,^69^ sunflower seed hull,^70^*Hevea brasiliensis* seed coat,^52^ coir pith,^71^ and date-pit.^75^ On the other side, a supreme decrease in color (91.84%) and COD (75.21%) was recorded, proving that bamboo waste can be applied in the extraction of AC for textile effluents treatment. In another research,^124^ the elimination effectiveness of a cypress cone-based AC was investigated for the COD, color, and turbidity removal from real textile wastewater. Based on the achieved results, the diminution in color, turbidity, and COD reduction was 80.4, 67.1, and 19%, respectively. Generally, it was reported that cypress cone-based AC also represents a promising material for the preparation of AC for industrial wastewater treatment. Table 6 represents a non-exhaustive list of AC diverted from different wastes and its adsorption capacity of various contaminants.

**Table 6:** Adsorption capacities of AC extracted from wastes utilized to remove diverse dyes and heavy metals

AC waste precursor	Contaminant	Adsorption capacity (mg g^−1^)	Ref.
Bamboo dust	MB	7.20	68
Groundnut shell		7.50	68
Coconut shell		8.16	68
Apricot stone		32.25	53
Apricot stone		36.68	53
Rice husk		37.57	68
Straw		42.60	68
Rice husk	Acid yellow 36	86.90	125
Sawdust		183.80	125
Mahogany sawdust	Direct green B	327.90	126
Mahogany sawdust		518.00	126
Bamboo dust	Pb^2+^	2.15	82
Nut shells	Cd^2+^	104.17	85

Additionally, numerous researchers have reported the great performance of AC for heavy metals removals, such as nickel, cobalt, cadmium, copper, and chromium.^127,128^ The adsorption of Cd^2+^ and Hg^2+^ on ozonized AC has been reported.^129^ The authors concluded that electrostatic forces were leading for the Cd^2+^ adsorption and the dispersive forces were dominant in the Hg^2+^ adsorption. Kadirvelu *et al.*^128^ revealed that the AC extracted from agricultural wastes can effectively eliminate Hg^2+^ and Ni^2+^ from aquatic solutions in a short time with high removal rates. A study was examined to assess the adsorption rate of CAC and bamboo dust carbon (BDC) to exclude Pb^2+^ ions from metal industry effluent.^82^ The achieved results exposed BDC as a remarkable adsorbent for the deduction of Pb^2+^ ions and can be an alternative adsorbent to CAC to remove metal ions from water.

#### 3.2.1 Agricultural waste adsorbents

Many cost-effective agricultural waste materials such as grass waste,^35^ orange peel,^130^ yellow passion fruit peel,^36,131^ garlic peel,^132^ pineapple stem,^133^ phoenix tree leaf,^134^ and peanut hull,^135^ can be used as adsorbent materials for dye removal. Anantha *et al.* reported the preparation of Bermuda grass (BG), which was treated by microwaves to remove MB from wastewater.^136^ In a microwave oven, raw BG with 3% NaOH was treated for 10 min. The obtained product was washed many times with water until it reached neutral pH. Then, the mixture was dried at 50 °C. The prepared sample showed a 171.25 mg g^−1^ maximum adsorption capacity. While, for removing blue 221 dye from aqueous solutions, mango peel as natural adsorbent material was used and compared its activity with ZnO NPs. Mango peel exhibited a maximum adsorption capacity of about 476.19 mg g^−1^ with respect to 133.33 for ZnO NPs.

Agricultural wastes are considered an inevitable source of undesirable solid waste. However, they are abundant in nature and require little processing to be reused as low-cost sorbent materials. The optimal use of agricultural wastes is represented in many treatment processes based on the adsorption process.^51^ Numerous researchers recorded the feasibility of using agricultural waste to remove dyes and metal ions.^7,24,49,73,137,138^ Many agricultural waste materials are used as sorbent materials for the elimination of different types of dyes from water at diverse operating conditions; grass waste,^35^ orange peel,^130^ yellow passion fruit peel,^36^ mango seed,^131^ garlic peel,^132^ pineapple stem,^133^ phoenix tree leaf,^134^ peanut hull^135^ and palm kernel shell.^74,139^ These materials are obtainable and may have potential as sorbents owing to their physicochemical characteristics and negligible price. Various organic compounds (lignin, cellulose, and hemicellulose) with polyphenolic groups are useful for binding dyes through different mechanisms.^24^ The efficiency of utilizing spent green tea leaf powder waste (SGTLP) for the decolorization of textile wastewater was reported in literature.^140^ The recorded results revealed high color removal efficiency at acidic medium and elevated temperature. From the viewpoints of waste recycling, the use of SGTLP has a remarkable effect on colour adsorption and representing an economical attractive alternative for textile wastewater treatment.

Batzias *et al.*^141^ reported great adsorption performance of beech saw dust as an economical sorbent material for MB and basic red 22 removals. Additionally, the authors verified the potential of the beech saw dust adsorbent after treating with CaCl_2_, which advanced the adsorption properties of the original material. In ref. 132, the application of garlic peels (GP) has been investigated to remove MB dye from an aqueous solution. The supreme adsorption capacities were 82.64, 123.45, and 142.86 mg g^−1^ at 303, 313, and 323 K, respectively. The authors concluded that GP could potentially apply basic dyes removal attributable to the great existence of polar functional groups. The adsorption capacity of some agriculture waste sorbents for the exclusion of numerous dyes and heavy metals is itemized in Table 7.

**Table 7:** Different agriculture waste adsorbents with their adsorption capacity of dyes and metal ions

Waste precursor	Contaminant	Adsorption capacity (mg g^−1^)	Ref.
Phoenix tree leaf	MB	89.70	134
Pineapple stem		119.05	133
Mango seeds		142.86	131
Garlic peel		142.86	132
Broad bean peel		192.70	142
Alfa stems		200.00	33
*Citrus limetta* peel		227.30	37
Jackfruit peel		285.71	143
Guava leaf powder		295.00	144
Pomelo (*Citrus grandis* peel)		344.83	145
Papaya seeds		555.56	34
Wheat shells		21.50	146
Yellow passion fruit peel		6.80	36
Coir pith	CR	6.72	71
Orange peel	Acid violet 170	19.88	130
Mixture almond shell	Direct red 80	20.50	147
Peanut hull	Reactive black 5	55.50	135
Olive stones	Cd^2+^	0.58	148
Potato peels	Cr^6+^	13.09	149
Mango leaves	Cu^2+^	15.77	150
Peanut shell	Cu^2+^	25.30	151
Peanut shell	Cr^3+^	27.86	151
Sunflower leaves	Cu^2+^	89.37	152
Orange peel	Pb^2+^	204.50	153

In another study, Feng *et al.*^154^ indicated that the chemically modified orange peels have enhanced contaminants' adsorption more than the unmodified orange peels. The obtained results indicated a maximum adsorption capacity of the modified orange peels for Pb^2+^, Cd^2+^, and Ni^2+^ ions removal as 476.1, 293.3, and 162.6 mg gm^−1^, respectively, at a contact time of 150 min and pH 5.5. Also, Lugo *et al.*^57^ investigated the elimination of Cr^3+^ and Fe^3+^ from aquatic solutions onto orange peels adsorbent. The results revealed that the optimum adsorption capacity of orange peel for Cr^3+^ and Fe^3+^ was 9.43 and 18.19 mg g^−1^, respectively. In conclusion, agricultural waste materials demonstrated efficient, low-cost adsorbents for contaminants removal.

#### 3.2.2 Industrial waste materials

There are many types of industrial waste sorbent materials that can be used for dye removal, including fly ash, red mud, blast furnace sludge, and metal hydroxide sludge. Wang *et al.* used both thermally and chemically treated fly ash and red mud to remove MB.^155^ Electrostatic precipitators-assisted raw fly ash (FA) sample was collected from the Western Power, Australia. One portion of the sample was thermally treated overnight at 800 °C, and the other portion was chemically treated at room temperature using (1 N) HNO_3_ solution for 24 h. Then, the treated sample was filtrated, washed, and dried overnight.

While, waste red mud (RM) was obtained from Worsley Alumina, Australia's downstream slurry pond. By filtration of the slurry, solid materials were collected and then dried at 110 °C overnight. Two samples were prepared by heat treatment at 800 °C overnight and acid-treatment using (1 N) HNO_3_ solution at room temperature for 24 h. Post-treatment, the latter sample was filtered, purified, and dried overnight at 110 °C.

Daily, enormous quantities of industrial solid wastes are produced without a proper treatment method. Recently, searching for an effective environmental method to reuse these wastes has become a concern to many researchers. On account of the great ability of these materials to adsorption, in addition to being inexpensive, numerous works have shown the ability of these wastes to adsorb dyes and heavy metals from wastewaters, as presented in Table 8. There are many types of industrial waste sorbent materials, such as fly ash, red mud, blast furnace sludge, metal hydroxide sludge, *etc.* Coal fly ash has been evaluated^156^ for real textile effluent treatment. The obtained results revealed that coal fly ash is efficient for the textile effluent decoloration up to 83.00% and for COD removal up to 61.11% at acidic medium (pH ≤ 2), temperature >20 °C, contact time of 3–5 min and adsorbent dose of 12–40 g L^−1^. The adsorption capability of metal hydroxide sludge has been assessed for the removal of azo reactive dyes.^157^ The authors revealed that metal hydroxide sludge was a positively charged adsorbent with a great adsorption capacity of up to 62 mg g^−1^. Khan *et al.*^158^ have examined iron oxide activated red mud (IOARM) for cadmium elimination from aqueous solution. The optimum operating conditions were recorded as pH 6.0, contact time 90 min, dose 6.0 g L^−1^, initial concentration 400 mg L^−1^, and temperature 300 K. The obtained results revealed that IOARM is a capable adsorbent for cadmium removal from water based on natural conditions.

**Table 8:** Maximum sorption capacities of various industrial waste adsorbents used for the removal of several contaminants

Waste precursor	Contaminant	Adsorption capacity (mg g^−1^)	Ref.
Red mud	Reactive black 5	35.58	159
	Acid blue 113	83.33	159
	Phosphate	0.58	160
	Cu^2+^	5.35	161
Coal fly ash	Cd^2+^	18.98	162
	Cu^2+^	20.92	162
	Cu^2+^	48.80	163

#### 3.2.3 Natural materials

Another class of cost-effective materials is natural materials such as clay, zeolite, and siliceous materials, which possess proper surface properties and large surface area, and eco-friendly nature. Aguiar *et al.* evaluated the removal of Acid Blue 25 (AB25) and Remazol Violet 5R (RV5R) reactive dyes by natural bentonite-derived porous clay heterostructures (PCHs).^32^ Three samples were prepared and compared, silica-supported PCH (Si-PCH) natural bentonite and silica-zirconia supported PCH (SiZr-PCH). To prepare PCH samples, hexadecyltrimethylammonium bromide (HDTMBr) (9 g) was used to treat Na-montmorillonite (2.5 g) in the presence of *n*-propanol (100 mL) for 3 days under continuous stirring. Then, the mixture was filtered and washed with D.W. to remove any HDTMBr residual. After that, using D.W. (250), the solid was redissolved for 34 h. Then, *n*-propanol dissolved hexadecyl amine solution (25 mL) containing 0.9 g (co-surfactant) was added to the above solution and left for 24 h under stirring. To form Si-pillars between montmorillonite adjacent layers, TEOS solution (11.1 mL) was added. While, silica-zirconia pillars with Si/Zr = 5 molar ratio were formed by adding *n*-propanol-dissolved solution containing both TEOS (9.4 mL) and zirconium propoxide (2.25 mL). Then, obtained gels after 72 h of stirring were collected, filtered, and washed with D.W. and ethanol, then dried in air at 60 °C. Finally, calcination at 550 °C was performed to remove the employed surfactant for 6 h.

**Fig. 16:**
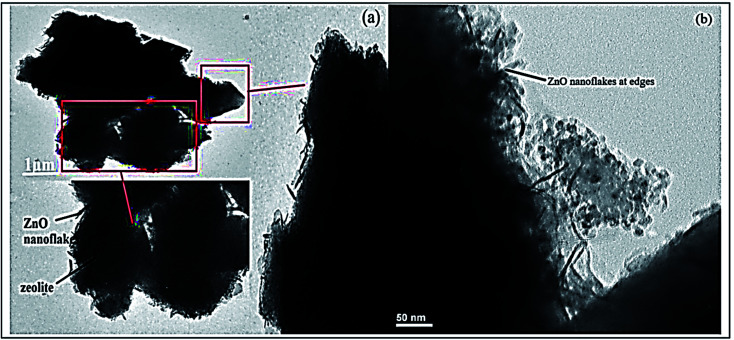
TEM analysis of the prepared ZnO@Ze nanocomposite at different magnifications (a and b), adapted with permission from ref. 164, copyright Elsevier, 2019.

While, the efficient removal of Congo red dye was reported by Madan *et al.*, using high-silica zeolitic particles (core) functionalized with ZnO nanoflakes (shell) prepared by precipitation method.^164^ In brief, KOH (1.48 g) was added to (65 mL) methanol solution, which was added drop-by-drop to another solution containing Zn(CH_3_COOH)·2H_2_O (2.95 g) dissolved in (125 mL) of methanol, the resultant solution was left under constant stirring for 2.5 h. After washing the obtained precipitate with methanol, it was immersed in a nanoparticle seeding solution containing methanol, *n*-butanol, and chloroform. After that, it was dried at 350 °C. Furthermore, prepared solutions of hexamethylenetetramine (1% w/v), polyethylenimine (3.3% v/v), and zinc nitrate (2.6% w/v) were obtained by dissolution in water (30 mL) to prepare ZnO nanoflakes on the obtained zeolite (1.5 mL) of ammonia solution was added to a mixture of (30 mL) PEI solution, (50 mL) HMTA solution, and (30 mL) zinc nitrate solution. Then, (1 g) of previously prepared zeolite powder was added, and the mixture was left under stirring at 95 °C for 4 h. Finally, the obtained mixture was filtered, washed many times with D.I.W., and activated for 30 min at 450 °C. Fig. 16 presents TEM analysis of the prepared nanocomposite, confirming ZnO nanoflakes distribution on zeolite particles' surface.

Recent trends in low-cost sorbent materials have led to a proliferation of studies evaluating natural materials for adsorption. These natural materials are eco-friendly, economical, abundant, and have the dramatic ability for adsorption.^165^ The high adsorption ability of natural materials is considered a sequence of their great surface area and the net negative charge on the minerals structure, which bounces the competence to adsorb positively charged contaminants.^48^ Different natural materials such as clay, zeolite, and siliceous materials have been proposed for dyes and heavy metals removal from wastewater (Table 9).

**Table 9:** Adsorption capacities of various natural materials used for the removal of several contaminants

Adsorbents	Contaminant	Adsorption capacity (mg g^−1^)	Ref.
Alunite	Reactive red 124	2.85	171
Zeolite	Basic dye	55.86	172
Clay	MB	300.00	173
Clay	Methyl green	427.00	173
Clay	Methyl violet	526.00	173
Clay	Neutral red	567.00	173
Zeolite	Mn^2+^	76.78 (mmol kg^−1^)	174
Zeolite	Zn^2+^	133.85 (mmol kg^−1^)	174
Zeolite	Cu^2+^	141.12 (mmol kg^−1^)	174
Zeolite	Co^2+^	244.13 (mmol kg^−1^)	174

**Fig. 17:**
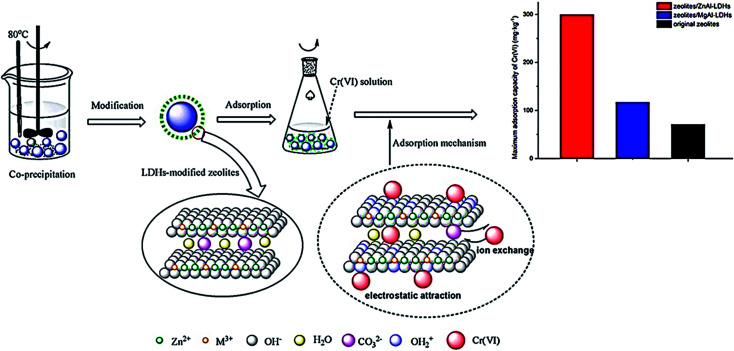
Removal difference of Cr^6+^ by modified zeolites coated with MgAl and ZnAl-layered double hydroxides, adapted with permission from ref. 170, copyright, Elsevier, 2021.

Clay minerals such as bentonite, kaolinite, and diatomite demonstrate a strong affinity for cationic and anionic dyes. Nevertheless, the adsorption ability for basic dye is much better than for acid dye due to the ionic charges on the dyes and clay character. The bentonite capacity for the removal of basic dyes was demonstrated to be 360.5 mg g^−1^ by the authors in ref. 166. Similar results have been reported in ref. 167 basic blue 9 dye removal. Moreover, the adsorption capacity of Fullers Earth and CAC was compared in ref. 168 for MB dye from an aqueous solution. The authors revealed that Fullers Earth is more efficient than CAC. Sdiri *et al.*^169^ studied the removal of Cu^2+^ and Zn^2+^ based on natural clay from diverse aqueous systems, as shown in Fig. 17. The obtained results presented that respectable adsorption capacities can be reached under pH 6, 1 h, and 1 g L^−1^ adsorbent dose at room temperature. In conclusion, natural clay adsorbent showed potential adsorption for Zn^2+^ ions removal than Cu^2+^ ions.

#### 3.2.4 Biosorbents

**Fig. 18:**
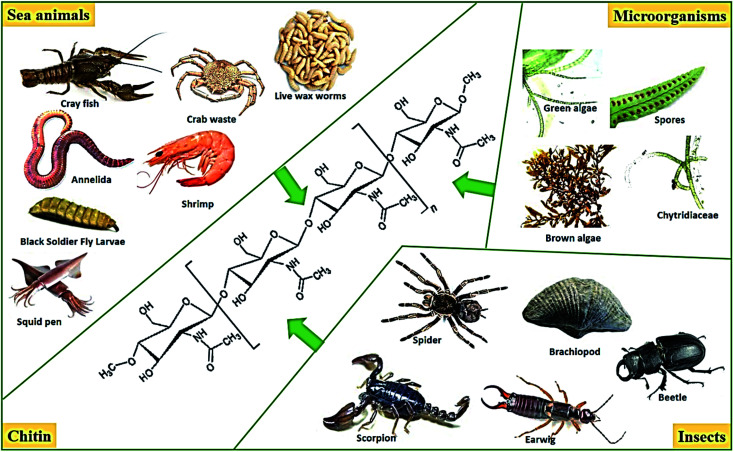
Sources of chitin.

Recently, there has been increasing interest in functionalized materials that bind with dyes and heavy metal ions in aqueous solutions.^175,176^ A common example of these materials is the biosorbent materials available in various forms such as chitin, chitosan, peat, yeasts, fungi, or bacterial biomass. Chitin and chitosan have copious surface areas and can be classified as sustainable, biodegradable polymers. In particular, chitosan can be obtained from crustacean chitin extracted from the crust of sea animals and insects and found in various microorganisms, as shown in Fig. 18.

The great adsorption capability of biosorbents is heavily reported. The adsorption rate of some biosorbents to exclude many dyes and metal ions is listed in Table 10. Fu and Viraraghavan demonstrated the *Aspergillus niger* (dead fungal biomass) adsorption performance in diverse studies.^177–180^ The studies showed a notable performance for *Aspergillus niger* fungi application as an adsorbent for dyes removal. Aksu *et al.*^181^ reported a great adsorption capacity (588.2 mg gm^−1^) for reactive black 5 dye using *Rhizopus arrhizus* biomass. Also, Waranusantigul *et al.*^182^ reported the applicability of using *Spirodela polyrrhiza* biomass to exclude methylene blue dye. Chitosan has an extensive consideration as a competent adsorbent thanks to its great contents of amino and hydroxyl functional groups compared to AC. It showed great potential for adsorption of varied contaminants, for example, dyes and heavy metals. The adsorption on chitosan has been reported for acid dyes removal by the authors in ref. 183. They concluded maximum adsorption capacities of chitosan for acid orange 12 (973.3 mg gm^−1^), acid orange 10 (922.9 mg gm^−1^), acid red 73 (728.2 mg gm^−1^), and acid red 18 (693.2 mg gm^−1^). Cardoso *et al.*^79^ compared *Spirulina platensis* microalgae (SP) and CAC to absorb reactive red 120 (RR-120) dye from aqueous solutions. The determined adsorption capacities of RR-120 dye have been reported 482.2 and 267.2 mg gm^−1^ for the SP and CAC adsorbents, respectively, at pH 2 and 298 K. It was also investigated by the authors that SP and CAC adsorbents presented respectable performance, 97.1 and 96.5%, respectively, of a dye mix at elevated saline concentrations. The removal of cadmium as a toxic heavy metal was assessed using CAC, chitosan and chitosan/AC composite adsorbents.^83^ The obtained results by the authors have revealed the maximum adsorption capacities of CAC, chitosan and chitosan/AC composite as 10.30, 10.00, and 52.63 mg gm^−1^, respectively at optimum operating conditions pH 6 and adsorbent dose 6 g L^−1^. Chitosan-based nanofibres were also evaluated for 3-methyl-4-nitrophenol removal from aqueous solutions. The findings revealed significant adsorption capacity reached 90% for at least 3 consecutive cycles.^184^

**Table 10:** Maximum sorption capacities of various biosorbent materials used for the removal of several contaminants

Biosorbent	Contaminant	Maximum adsorption capacity (mg g^−1^)	Ref.
Algae (green algae *Ulva lactuca*)	Dye (methylene blue)	40.2	189
Algae (brown algae *Cystoseira barbatula* Kutzing)	Dye (methylene blue)	38.61	190
Fungi (*Aspergillus niger*)	Heavy metal (Cu^2+^)	15.6	191
Fungi (*Aspergillus niger*)	Heavy metal (Pb^2+^)	34.4	191
Fungi (*Aspergillus niger*)	Heavy metal (Cr^6+^)	6.6	191
Bacterial biomass (*Bacillus thuringiensis*)	Heavy metal (Ni^2+^)	45.9	192
Chitosan	Acid orange 12	973.3	183
Chitosan	Acid orange 10	922.9	183
Chitosan	Acid red 73	728.2	183
Chitosan	Acid red 18	693.2	183
Chitosan	Acid green 25	645.1	183

Cellulose based adsorbents (CBA) are considered promising and cost-effective for removing dyes and metal ions from water.^185,186^ Because of the abundance of hydroxyl, carboxyl, and phenolic groups throughout the backbones of cellulose, CBA have the ability to bind contaminants effectively. Moreover, the porous structure of cellulose which allows active agents to disperse inside the substrates.^187,188^ Li *et al.*, 2020 (ref. 186) applied the liquid phase reduction technique to load sawdust cellulose on zero-valent iron to remove arsenic and Cr^3+^ ions from aqueous solution. The results revealed high adsorption capacities reached 111.37 and 268.7 mg g^−1^ for arsenic and Cr^3+^ ions, respectively. Porous magnetic cellulose/Fe_3_O_4_ beads was also investigated for MB and rhodamine B (RhB) dyes removal from aqueous solution.^185^ The maximum adsorption capacity reached 1186.8 and 151.8 mg g^−1^, respectively. Li *et al.*^188^ developed an innovative composite adsorbent based on dissolved cellulose fibres and microfibrillated cellulose modified by nano-sized CaCO_3_ as a pore forming agent for MB dye removal. The results showed maximum adsorption capacity of MB at 303 mg g^−1^. Moreover, fine aminated cellulose/montmorillonite mesoporous composite beads achieved great adsorption rate for auramine O dye reached 1336.2 mg g^−1^ at 55 °C.

### 3.3 Metals and metal oxide-based nanomaterials

#### 3.3.1 Metal-based materials

**Fig. 19:**
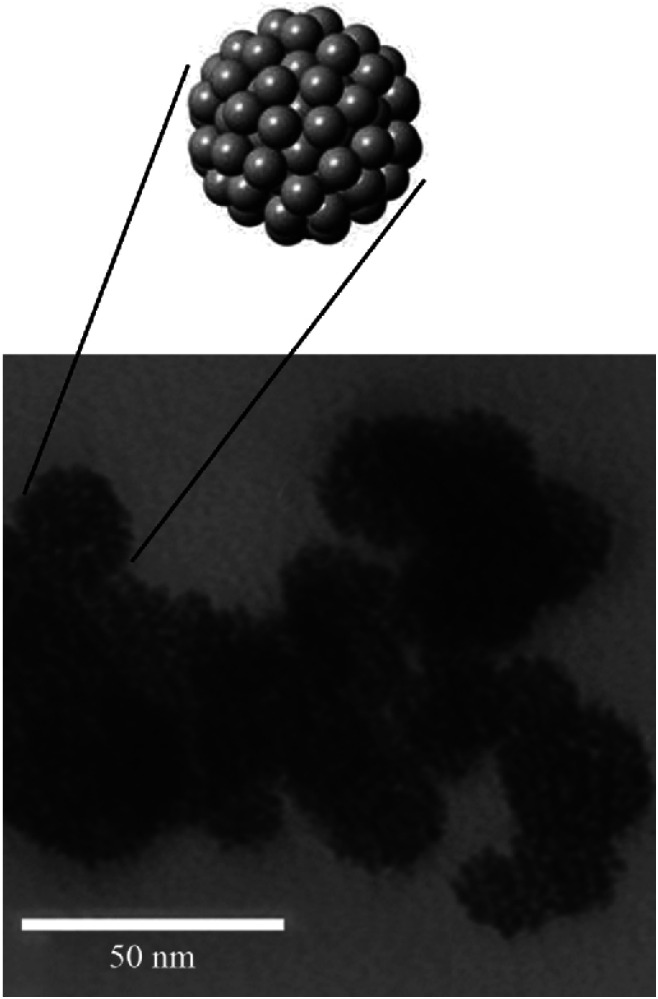
TEM micrograph of the prepared zero-valent iron NPs, adapted with permission from ref. 194, copyright, Elsevier, 2018.

Due to their surface reactivity, large surface area, and small particle size, zero-valent metals such as (iron, copper, and aluminum) are extensively investigated for their potential adsorption abilities. Marcelo *et al.* prepared zero-valent copper nanoparticles to remove blue 4 reactive dye using a chemical reduction method.^193^ Firstly, copper sulfate solution was prepared by dissolving copper(ii) sulfate pentahydrate (8.85 g) in (50 mL) ethanol–water mixture with (4 : 1, v/v) ratio *via* stirring for 15 min. Then, sodium borohydride (1.10 M) reducing agent was dipped to the mixture under constant stirring with (1–2 drops per second) rate. Finally, the sample was filtered, washed with water and ethanol, and dried under a vacuum. While Ebrahiminezhad *et al.* prepared ultra-small clusters of zero-valent iron nanoparticles by green method to remove MO.^194^ The mean diameter of the prepared particles was about 19 nm, as shown in Fig. 19. (9 mL) of Mediterranean cypress (*Cupressus sempervirens*) leaf extract was vigorously stirred in a (50 mL) round bottom flask at ambient temperature. Then, 1 mL of FeCl_3_·6H_2_O (1 M) solution was injected into the flask under constant stirring for 24 h. After that, the formed black precipitate was collected by centrifugation, followed by D.I.W. washing three times to remove reaction residuals. Finally, the collected powder was dried at 50 °C in the oven.

#### 3.3.2 Metal oxide-based materials and hybrid composites

**Fig. 20:**
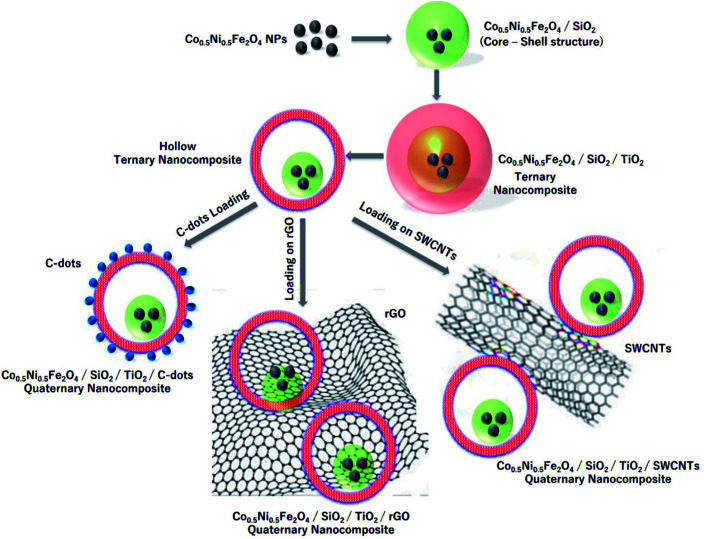
Schematic presentation of the steps used to prepare CNFST composite matrix loaded with C-dots, rGO, and SWCNTs, adapted from ref. 204 with copyright permission from Elsevier, 2020.

Most recent reports investigated the potential adsorption capacity of bare metal oxide-based nanomaterials and their hybrids, prepared by various methods to remove various dyes, as summarized in Table 11. Numerous nanomaterials have been studied and developed to remove serious contaminants such as heavy metals and color from industrial wastewaters with low cost and high efficiency. The admirable characteristic of these materials is their nanosized scale and their large surface area associated with their small size. Moreover, the exceptional electron conduction properties could offer sorbent nanomaterials with excellent evaluation in heavy metals and dyes removal from real industrial wastewaters. For example, Abd Elkodous *et al.* reported a new TiO_2_-based nanocomposite decorated with different carbon materials to remove chloramine-T from water, as shown in Fig. 20. In this review, nanomaterials based on carbon, silica, zero-valent metal, metal-oxide, and nanocomposites were systematically reviewed along with their applications in the removal of heavy metals and dye from industrial wastewater.

**Table 11:** Adsorption capacities of metal oxide nanomaterials for various dyes and heavy metal ions removal

Metal oxide	Preparation method	Tested dye/heavy metal	Capacities (mg g^−1^)/removal efficiency (%)	Size range/crystallite size (nm)/surface area (m^2^ g^−1^)	Ref.
MgO	Green synthesis using *Tecoma stans* (L.) extract	Congo red (CR) and crystal violet (CV)	142.17–150.49	SR: from 20 to 50	195
	Microwave-assisted combustion method	CR and trypan blue	136–132	Average particle size 18	4
	Precipitation method	Reactive blue 19 (RB19), reactive red 195 (RR 195), CR, MB, and rhodamine B	549.45 to RB19, 348.43 to CR, and 442.48 to RR195	CS: 37/SA: 154.85	196
	Green synthesis using *Aspergillus niger*	Textile wastewater containing Cr, Co, Pb, Cd, and Ni, heavy metals	94.2% ± 1.2%, 63.4% ± 1.7%, 72.7% ± 1.3%, 74.1% ± 1.8%, and 70.8% ± 1.5%	SR: nano-rectangular (18.6–27.6) and nano-rods (30–85)	197
TiO_2_	Sol–gel/green synthesis using *Tridax procumbens* leaf extract	MO	273.37 at 303 K	—	198
CdS/TiO_2_ decorated carbon nanofibers	Electrospinning method	MB reactive black 5, and reactive orange 16	95% removal after 5 min	—	199
MgFe_2_O_4_–TiO_2_@GO	Ultrasonication method	MB	99% removal at 50 ppm/0.05 g conc. and adsorbent dose	SR: from 20 to 30 – SA: 58.48	200
AC/TiO_2_/chitosan	Impregnation method	Safranin O	357.14	SR: 29.83–50 – SA: 834.477	201
Al_2_O_3_/GO-based cellulose	Green synthesis	Fluoride	5.34 in pH 5 after 120 min	CS of Al_2_O_3_: 5.52	202
ZnO	Green synthesis	CR – malachite green	92.30% after 120 min to 90.7% after 90 min	MD: 44 – CS: 37.86 – SA: 63.09	203

#### 3.3.3 Metals-based nanomaterials

**Fig. 21:**
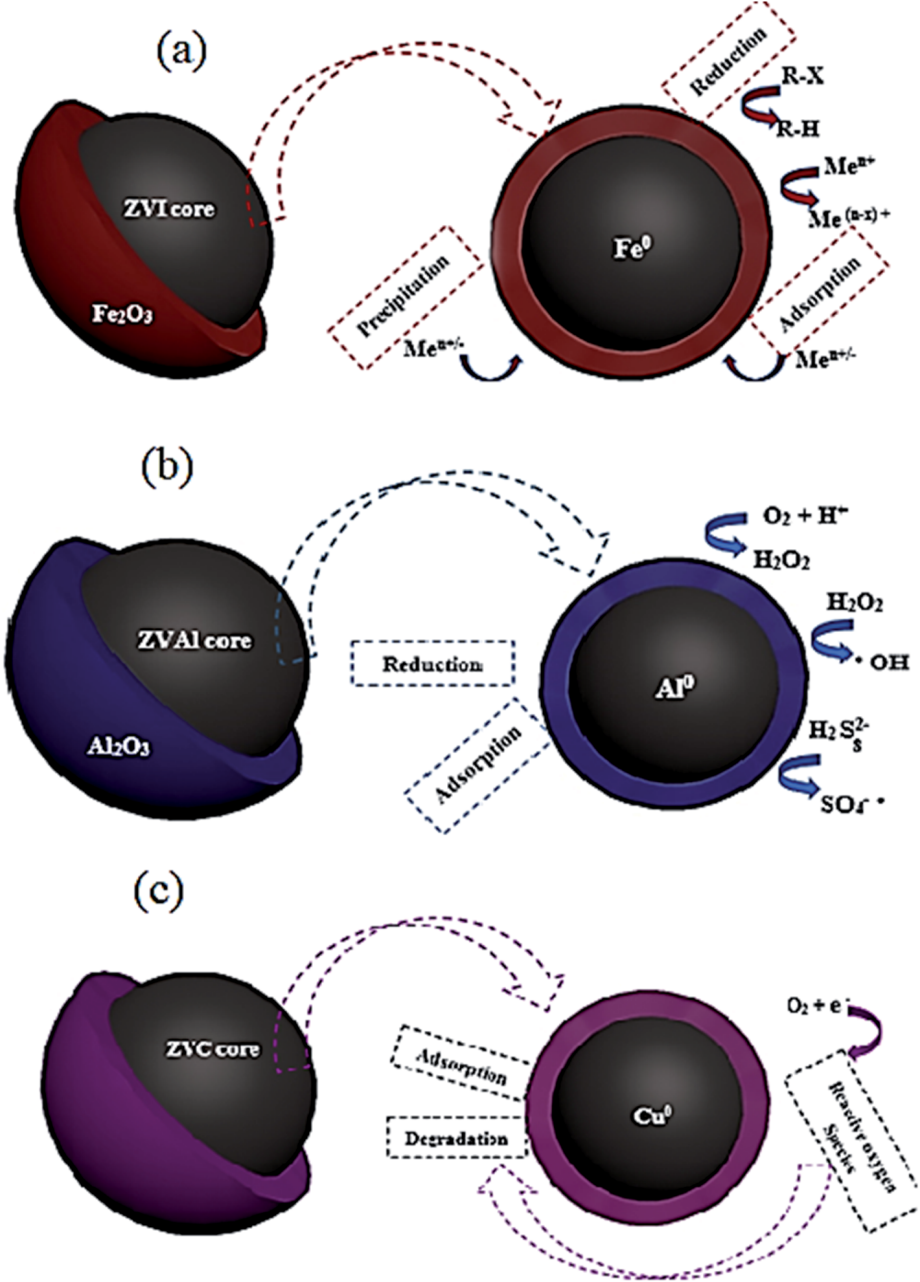
Nano zero-valent iron (a) aluminum (b), and copper (c) core–shell structures with different mechanisms of contaminants removal.

In recent years, zero-valent metal nanomaterials such as zero-valent iron (ZVI), zero-valent copper (ZVC), zero-valent aluminum (ZVAl) have demonstrated potential for use in industrial wastewater treatment.^25,86,205^ The composition of these zero-valent metal nanomaterials enhanced their capabilities to deal with different types of pollutants. Zero-valent metals, such as ZVI and ZVC are good electron donors. The pollutant molecules accept electrons from the metals and convert them into transitional products when combined with H^+^. Nano zero-valent iron (nZVI) was reported to have an excellent degradation capability towards dyes and organic substances.^206^ Moreover, the high evaluation on the exclusion of metal ions.^207^ It has received increased consideration as a novel sorbent material for treating diverse kinds of dyes and numerous types of metal ions.^208^Fig. 21a shows the nZVI composition of Fe^0^ and ferric oxide coating shell with different mechanisms of contaminants removal. Nano zero-valent iron particles can provide a large surface area full of abundant reactive sites besides the ferric oxide shells' extensive reactive and electrostatic interaction. Moreover, the high reduction ability resulting from the presence of Fe^0^ can diminish different types of pollutants.

In early research, the disperse red 1 azo dye adsorption on nZVI has been evaluated.^209^ About 98% color removal was recorded in a fairly short time 10 min. Also, the capability of adsorbing MB basic dye was studied at optimum operating conditions of pH 9.5, adsorbent dosage 0.5 g L^−1^, and contact time of 1 min.^208^ The extreme adsorption rate of nZVI towards MB was 208.33 mg g^−1^, demonstrating a promising adsorbent for dying effluents treatment. In another study,^206^ the authors examined the removal of Cr^6+^ on bentonite-supported nano zero-valent iron (B-nZVI) and unsupported nZVI. The B-nZVI showed great removal results 99% of Cr^6+^ at 250 rpm and 35 °C with 50 mg L^−1^ initial concentration of Cr^6+^. In a comparative study,^2^ the concert of using nZVI and AC was examined for the removal of color from real textile wastewater. The results showed the enhanced color removal for the two examined adsorbents reached 84% for the AC and 80% for the nZVI at same operating conditions 0.8 g L^−1^ dose and 20 min contact time. A scaling up treatment study was investigated by Badawi *et al.*^210^ to assess the viability of using nZVI in a combined treatment system for the color removal from real textile wastewater. The results revealed maximum colour removal reached 98.4% at 0.8 g L^−1^ dose. The separation and recycling process was applicable depending on the magnetic properties of the nano iron particles. Lately, nano zero-valent aluminum (nZVAl) has gained an increasing interest in the field of wastewater treatment through its abundant surface area and high surface reactivity.^211^ It was observed that nZVAl can generate hydroxyl and sulfate radicals, eliminating non-biodegradable contaminants in a short reaction time from water.^212^Fig. 21b shows the core structure on nZVAl particle with different mechanisms of contaminants removal. Zero-valent aluminum is considered efficient sorbent material and has a higher catalytic activity to treat industrial wastewaters.^211,212^ The superior performance of nZVAl in removing dyes and heavy metals from wastewater has been investigated by a numerical study.^213–215^ Guo *et al.* found that ZVAl revealed an exceptional ability for the acid orange 7 (AO7) degradation under ultrasonic irradiation.^213^ It was reported that the decolorization rate improved with the increase of ZVAl dose and power density. About 96% removal rate was reported for the AO7 dye at pH 2.5, 30 min contact time, 20 mg L^−1^ initial dye concentration, 2 g L^−1^ ZVAl dose and 20 kHz with 300 W ultrasound. In another study,^215^ the authors have studied the treatment of textile effluent by advanced oxidation processes (AOPs) based on ZVAl. Experiments were conducted for the removal of COD, color and ammoniacal nitrogen removal by using different combinations with ZVAl; ZVAl/O_2_, ZVAl/Fe^3+^/O_2_, ZVAl/Fe^3+^/O_2_/H_2_O_2_ and ZVAl/Fe^3+^/O_2_/persulfate processes. Maximum removal results were recorded 97.9, 94.4 and 58.3% for COD, color and ammoniacal nitrogen removal, respectively 1 g L^−1^ ZVAl, 0.5 g L^−1^ Fe^3+^ and 6.7 g L^−1^ H_2_O_2_ in 3 h of reaction time. On the other side, the significant ability of activated ZVAl in eradicating Cr^6+^ from wastewater was testified by the authors in ref. 214. The exclusion rate of Cr^6+^ was enhanced with the increased load of acid washed ZVAl at low initial pH. About 98% removal rate was reported at optimum operating conditions: 180 min contact time, 0.4 g L^−1^ acid washed ZVAl dose at initial pH 2.0 for the eradication of Cr^6+^ from synthetic wastewater holding 20.0 mg L^−1^ Cr^6+^.

Improvement of the cost-effective sorbent material is essentially required to develop the band gap energy of the nanomaterials. Copper-based nanomaterials could offer an efficient, economical way for wastewater treatment thanks to their unique chemical, electrical, optical and thermal properties.^86^ Enormous researchers have examined nano zero-valent copper (nZVC) for the degradation of chemical pollutants,^216,217^ nitrate removal,^218,219^ phenol removal,^220^ and dyes degradation.^193,221^Fig. 21c shows the core–shell structure of the nZVC nanoparticles.

The effectiveness of using nZVC as a catalyst in combination with H_2_O_2_ and ultrasound irradiation for phenol degradation from an aqueous solution has been reported.^220^ The results exposed that the degradation of phenol is massive in the presence of nZVC with H_2_O_2_, which can be credited to the enhanced production of OH radicals in the medium. The maximum degradation rate extended to be 65% using 1 g L^−1^ of nZVC in the presence of 20 mM H_2_O_2_ after 60 min of sonication. In another study,^193^ the authors performed experimental investigations on the reactive blue 4 (RB4) dye degradation using nZVC. The degradation reaction was enhanced at low pH < 4 (acidic medium). About 90% removal rate was recorded in 10 min of reaction time. In the experiment of reusing the recovered ZVC nanoparticles, a slight loss of catalytic activity was observed, showing a degradation rate of 73% in the second cycle of the nanomaterial use.

#### 3.3.4 Metal oxide-based nanomaterials

Metal oxide nanoparticles have a superior ability to form adsorbents with great electronic properties. This is due to the decreased gap between the oxide particles and particles size, subsequent conductivity variation, and chemical reactivity.^222^ Metal oxide nanoparticles demonstrated advanced adsorption extent for dyes and heavy metal ions elimination thanks to the formation of ternary ligands.^223^ The adsorption efficiency of contaminants removal based on metal oxide nanomaterials commonly depends on pH. For instance, the heavy metal ions removal rate increases with pH due to the formation of metal complexes and electrostatic interactions, contingent on the chemistry among metal and wastewater or on the functional group nature. Also, rising the sites with negative charge increases the attraction forces among metal ions with a positive charge and the negative sites on sorbent materials. At low pH levels, adsorption occurred among the competing metal ions and H^+^ ions.^224^Table 12 summarizes various metal oxide nanomaterials utilized for the elimination of diverse dyes and metal ions.

**Table 12:** Extreme adsorption capacity for some metal oxide nanomaterials to deduce diverse dyes and heavy metals

Adsorbent	Contaminant	Adsorption capacity (mg g^−1^)	Ref.
MgO	Indanthren blue	86.50	63
MgO	Levafix fast red CA	92.16	63
MgO	Reactive brilliant red X3B	277.78	225
MgO	Congo red	303.03	225
TiO_2_	Cr^6+^	12.60	226
TiO_2_	Pb^2+^	21.70	227
TiO_2_	Ni^2+^	39.30	227
Al_2_O_3_	Pb^2+^	41.20	227
Al_2_O_3_	Ni^2+^	35.90	227
Al_2_O_3_	Cu^2+^	47.90	227
TiO_2_	Cu^2+^	50.20	227
MnO_2_/gelatin	Cd^2+^	105.10	228
Al_2_O_3_	Cd^2+^	118.90	227
TiO_2_	Cd^2+^	120.10	227
MgO	Cd^2+^	135.00	227
MgO	Pb^2+^	148.60	227
MgO	Cu^2+^	149.10	227
MgO	Ni^2+^	149.90	227
MnO_2_/gelatin	Pb^2+^	318.70	228
ZnO	Zn^2+^	357.00	229
ZnO	Cd^2+^	387.00	229
ZnO	Hg^2+^	714.00	229

### 3.4 Nanocomposite materials

Numerous nanomaterials have been studied and developed to remove serious contaminants such as heavy metals and color from industrial wastewaters with low cost and high efficiency. The admirable characteristic of these materials is their nanosized scale and their large surface area associated with their small size. Moreover, the exceptional electron conduction properties could offer sorbent nanomaterials with excellent evaluation in heavy metals and dyes removal from real industrial wastewaters. The synergistic effect of the nanocomposites also plays a critical role in the adsorption performance. Thus, many types of hybrid nanocomposite materials from metal/metal oxide (Fe/MgO),^230^ metal oxide/carbon materials (NiO/graphene),^231^ metal oxide/metal oxide (Fe_3_O_4_/MnO_2_, Co_3_O_4_/SiO_2_),^232,233^ metal oxide/carbon materials (MWCNTs-Fe_3_O_4_),^234^ and metal oxide/polymer materials (Fe_3_O_4_/polypyrrole)^235^ have been prepared and tested for dyes and metal ions removal. Abd Elkodous *et al.* reported a new TiO_2_-based nanocomposite decorated with different carbon materials to remove chloramine-T from water.^236^ MWCNTs-Fe_3_O_4_ nanocomposite showed high adsorption efficiency of 238.78 mg g^−1^ toward Hg^2+^.^234^ In addition, Co_3_O_4_/SiO_2_ showed a high ability to adsorb MB (53.87 mg g^−1^).^232^ Moreover, γ-Fe_2_O_3_–polypyrrole nanocomposite showed a high adsorption capacity of 464 mg g^−1^ of MB.^235^

## 4. Limitations and future challenges

Table 13 reviews the advantages and margins of various sorbent materials for industrial effluents treatment. It was reported that AC, CNTs, and graphene adsorbents could be considered to have the supreme ability for adsorption; however, it's costly to be regenerated. For the low-cost adsorbents, including agriculture, industrial waste adsorbents, natural materials, and biosorbents, large-scale continuous feed studies are needed to maximize the eco-friendly benefits of using these kinds of adsorbents. The nano-based adsorbents are considered great sorbent materials and are easy to regenerate but have some aggregation limits, affecting the adsorption rate.

**Table 13:** Advantages and limitations of various adsorbents for the treatment of industrial effluents

Adsorbent	Limitations	Advantages	Ref.
AC	Cost demanding through the regeneration process	Great adsorption performance and economically attractive when extracted from waste materials	21, 26, 44 and 56
Agriculture, industrial waste adsorbents, natural materials, and biosorbents	Needs more investigations in large scale continuous feed operations	Eco-friendly, high surface area, relatively high adsorption capacities, and low or zero cost demanding	7, 24 and 34
CNTs	Relatively expensive	Large surface area, great adsorption capacity, and easy to be modified	102, 237 and 238
Graphene	Relatively expensive	Exceedingly large surface area, great adsorption capacity, and easy to be modified	120, 223 and 239
Zero valent nanoparticle	Aggregation limits the adsorption rate	Small particle size, great surface area, low cost, degradation, great adsorption capacity, and easy to regenerate	209 and 218
Oxide nanoparticle	Aggregation limits the adsorption rate	Small particle size, fairly high efficiency, low cost, easy to separate when using iron oxide particles, easy to regenerate	229 and 240

## 5. Conclusions

This review has attempted to cover the common adsorption process operating conditions and an extensive range of non-conventional economic adsorbents to give an idea about the various types of low-cost sorbent materials used for dyes and metal ions removal from wastewater. Vitally significant characteristics for a satisfying adsorbent could be summarized as follow: (a) high porosity and great surface area can guarantee the availability of more adsorption sites, (b) great ion exchange ability, (c) abundant existing in nature and at enormous quantities, (d) possible to be of regenerated, (e) eco-friendly and (f) economic. ACs are commanding adsorbents that can powerfully eradicate several contaminants from polluted water; nevertheless, their limited utilization at large-scale applications due to high production costs and difficulty in regeneration. Numerous economic adsorbents have been reported to cover the need for low-cost and effective adsorbents that can eliminate dyes and metal ions from contaminated waters. For instance, agriculture, industrial waste materials, natural materials, and biomass absorbents. Numerous research studies extensively evaluate these sorbent materials; however, the large-scale application is still gapped. Nanotechnology is a novel way that emerged among the most efficient and economic adsorbents. However, it also needs to promote the application on a large scale to assess all technical, economic, and environmental aspects for use in wastewater treatment.

## Conflicts of interest

The authors declared no potential conflicts of interest concerning this article's research, authorship, and/or publication.

## Supplementary Material
